# Impact of a Digital Lifestyle Intervention on Diabetes Self-Management: A Pilot Study

**DOI:** 10.3390/nu14091810

**Published:** 2022-04-26

**Authors:** Maxi Pia Bretschneider, Jan Klásek, Martina Karbanová, Patrick Timpel, Sandra Herrmann, Peter E. H. Schwarz

**Affiliations:** 1 Department for Prevention and Care of Diabetes, Department of Medicine III, Faculty of Medicine Carl Gustav Carus, Technische Universität Dresden, Fetscherstrasse 74, 01307 Dresden, Germany; patrick.timpel@wig2.de (P.T.); sandra.herrmann@uniklinikum-dresden.de (S.H.); peter.schwarz@uniklinikum-dresden.de (P.E.H.S.); 2 Institute of Economic Studies, Faculty of Social Sciences, Charles University in Prague, Opletalova 26, 110 00 Prague, Czech Republic; klasekjan@gmail.com; 3 Department of Health, Faculty of Medicine, Masaryk University, Kamenice 5, 625 00 Brno, Czech Republic; martina.karbanova@med.muni.cz; 4 First Faculty of Medicine, Charles University in Prague, Kateřinská 32, 121 08 Prague, Czech Republic; 5 Paul Langerhans Institute, Faculty of Medicine, Technische Universität Dresden, Tatzberg 47, 01307 Dresden, Germany; 6 German Center for Diabetes Research (DZD), Ingolstädter Landstraße 1, 85764 Neuherberg, Germany

**Keywords:** diabetes mellitus type 2, self-management, digital health, HbA1c, lifestyle intervention, digital intervention, mHealth

## Abstract

The aim of this study was to provide preliminary evidence on the impact of the digital health application Vitadio on improving glycemic control in patients with type 2 diabetes mellitus. This was a 3-month, prospective, multicenter, open-label trial with an intraindividual control group. Participants received a digital lifestyle intervention. HbA1c levels were observed at 3 time points: retrospectively, at 3 months before app use; at baseline, at the start of usage; and 3 months after the start of use. In addition, changes in other metabolic parameters (fasting glucose, body weight, and waist circumference), patient reported outcomes (quality of life, self-efficacy, and depression), and data generated within the app (frequency of use, steps, and photos of meals) were evaluated. Repeated measures analysis of variance with the Bonferroni correction was used to assess the overall difference in HbA1c values between the intervention and the intraindividual control group, with *p* < 0.05 considered significant. Participants (*n* = 42) were 57 ± 7.4 years old, 55% male, and with a mean baseline HbA1c of 7.9 ± 1.0%. An average HbA1c reduction of −0.9 ± 1.1% (*p* < 0.001) was achieved. The digital health application was effective in significantly reducing body weight (−4.3 ± 4.5 kg), body mass index (−1.4 ± 1.5 kg/m^2^), waist circumference (−5.7 ± 15 cm), and fasting glucose (−0.6 ± 1.3 mmol/L). The digital therapy achieved a clinically meaningful and significant HbA1c reduction as well as a positive effect on metabolic parameters. These results provide preliminary evidence that Vitadio may be effective in supporting patient diabetes management by motivating patients to adopt healthier lifestyles and improving their self-management.

## 1. Introduction

Obesity and type 2 diabetes mellitus (T2DM) have reached the proportions of a worldwide pandemic. In Germany, 67% of men and 53% of women are overweight. One in four adults (23% of men and 24% of women) are obese [1]. In addition, more than 6.9 million people are affected by T2DM [2]. Results from a cross-sectional study of 2019 physician billing data showed that men and women with diabetes have a 3.7- and 3.8-fold higher prevalence of obesity, respectively, than those without diabetes [3]. This illustrates the relevance of obesity as a risk factor for the onset of T2DM. The increasing prevalence of obesity was described by an analysis of seven prospective cohort studies showing a weight increase of 0.25 kg a year over the last decade in middle-aged people [4]. Clearly, new approaches to prevention are urgently needed to stop this pandemic.

Lifestyle intervention includes dietary changes, weight reduction, increased physical activity, and also effective stress management. Current guidelines recommend lifestyle interventions for the primary prevention of metabolic syndrome and as a therapeutic component [5]. Several studies have already proven the effectiveness of lifestyle interventions in terms of weight loss and glycemic improvement [6,7]. The use of HbA1c has been integrated in studies to represent glycemic improvement [8,9]. Both the American Diabetes Association (ADA) guidelines [10] and the European Association for the Study of Diabetes (EASD) guidelines [11] classify the HbA1c value as the standard target for diabetes treatment. For patients with T2DM, lifestyle modifications in areas of nutrition, physical activity, and smoking cessation are the foundation of successful diabetes therapy. Furthermore, empowering patients with diabetes self-management and psychological assessment and improvement of diabetes-related distress, quality of life, and depression are key components of effective diabetes treatment [12,13,14]. To implement these principles of effective diabetes management and target the prevention of diabetes and obesity, digital health applications are increasingly important because they are not only effective but also cost-efficient [15]. Digital health apps can support patients in effective self-management and show progress in achieving treatment goals [16,17].

A variety of digital applications are already freely available to patients, but evidence of their effectiveness is mostly lacking. It is essential to close this gap to support the adoption of digital applications in health care [18]. In Germany, a structured approval process for digital health applications (DiGA) has been established by the Federal Institute for Drugs and Medical Devices (BfArM) [15]. In this assessment, information on product qualities, data protection, and positive healthcare effects are verified. After approval is granted, the DiGA is included in the DiGA directory, which lists all DiGAs that are reimbursed by health insurance.

Vitadio uses available evidence from the field of medical nutrition therapy, psychology, and behavioral intervention for patients with T2DM and obesity. The application is designed to improve diabetes treatment by empowering patients with effective self-management and lifestyle modification. The application is based on a multimodal therapy approach and combines strategies such as gamification, feedback, personalized goal setting, and social support, which are key components of a successful DiGA [15,19]. The aim of the EDDY trial was to provide preliminary evidence for Vitadio in patients with T2DM, with the intention of obtaining preliminary approval as a DiGA by the BfArM.

## 2. Materials and Methods

### 2.1. Study Design

**Figure 1:**
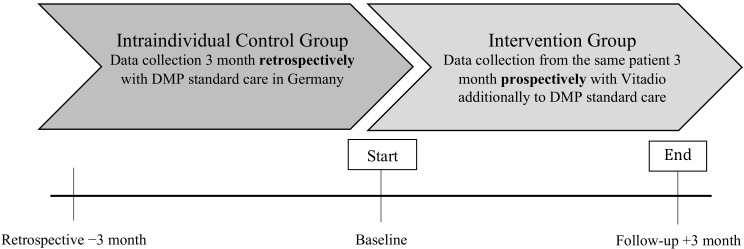
Study scheme.

A 3-month, prospective, multicenter, open-label observational study with an intraindividual control group was conducted to evaluate the impact of Vitadio on HbA1c in patients with T2DM (Figure 1). A follow-up period of 3 months was chosen, which is sufficient to demonstrate significant changes in HbA1c levels and metabolic parameters [20,21,22,23]. The study is registered in the German Clinical Trials Registry (DRKS00027392) and was approved by the Ethics Committee at the Technical University of Dresden (BO-EK-195032021) on 7 May 2021.

### 2.2. Participants

#### 2.2.1. Eligibility

Participants were included if they were over 18 years of age and were able and willing to use Vitadio as part of their diabetes management. Baseline HbA1c had to be in the following range: 6.5–11.0%. Participants must have been enrolled in the disease management program (DMP, a structured treatment program in Germany for patients with chronic diseases) for at least 6 months prior to the study to ensure consistency of care (standard of diabetes care) for the control group [24].

Principal exclusion criteria included using other apps for diabetes management, participating in a weight loss program during prior six months, using a diabetes app in the prior 12 months, treatment with use of an insulin pump or continuous glucose monitoring, and impairments which would seriously compromise the integrity of the study—including mental or psychic impairments.

#### 2.2.2. Recruitment

Participants were recruited via a Facebook campaign targeting all of Germany. Interested patients were redirected to the study webpage presenting a detailed description of Vitadio, the EDDY trial, i.e., study goals and duration, benefits and risks, inclusion and exclusion criteria, and remuneration. Patients registered for the EDDY trial on the website, accepted the online informed consent and privacy policy, and filled the eligibility assessment. The assessment consisted of six questions, including date of previous and upcoming physician visits. The information was checked again manually, and ineligible patients were notified by message. The eligible patients received a message with a unique identification code for the Vitadio app. Participants registered in the app and booked the onboarding call, which was conducted by a Vitadio assistant. The assistant confirmed eligibility criteria and provided information about the study procedure. When the eligibility was not confirmed, the participant was excluded from the study.

### 2.3. Study Procedures

The study was conducted in routine clinical care and ambulatory settings. At the beginning, patients were asked to fill out a baseline questionnaire in the app including demographic characteristics, diabetes duration, treatment, comorbidities, and metabolic parameters. In addition, patient reported outcomes (PRO) questionnaires on quality of life, self-management, and depression were sent electronically to participants at two time points (at baseline and 3-month follow-up of Vitadio use). The HbA1c values were documented in the case report form (CRF) at 3 time points (i.e., retrospectively, at 3 months before Vitadio use; at baseline; and at 3-month follow-up of Vitadio use). The CRF was completed and signed by the physician or a member of the care team or by the patient. At the end, participants were asked to submit the CRF and complete a final questionnaire also including metabolic parameters. Additionally, data generated by the app were collected, including self-reported metabolic parameters (e.g., weight, waist circumference, and fasting glucose), as well as frequency of use, steps, meal photos, and in-app questionnaires on self-efficacy. Successful completion of the study occurred when a participant completed all visits of the study. The participants received a financial reward of 30 EUR in the form of an Amazon voucher if they submitted all study data (i.e., HbA1c at all 3 time points and patient-reported outcomes at baseline and 3 months). Participants who withdrew consent or participants who were withdrawn by the investigator did not proceed to the final measurement.

### 2.4. Intervention

Vitadio is a digital care program designed to empower patients with effective self-management and lifestyle change. It consists of a three-month intensive phase followed by a sustained phase. The mobile application guides patients throughout the program using a system of daily tasks and automated messages. Patients follow educational courses, including topics ranging from motivation to diet, physical activity, sleep hygiene, mental wellbeing, and social aspects of life with diabetes. Personal weekly goals help to select relevant habits and track them daily. The Vitadio app enables monitoring of metabolic (e.g., body weight, waist circumference, glycemia) and lifestyle (e.g., steps, diet, mood) parameters. To track dietary habits, the patients can use a feature designated to upload photos of their meals. The program is enhanced by a set of communication features employing human support. To ensure patient safety and enhance effective use of the program, a personal advisor is available by chat to answer patient questions. To improve adherence, patients can participate in a peer support group. Vitadio complements therapy set by a physician and is certified as a class I medical device. Examples of the user interface of the Vitadio app can be found in Appendix A.

### 2.5. Comparator

The Control group consists of retrospective observations of the same participants from the intervention group. Therefore, HbA1c values measured 3 months before the start of the study were collected retrospectively. The control group received standard diabetes care as defined by the German diabetes disease management program and provided by diabetes specialists and/or general practitioner in Germany.

### 2.6. Outcome Measurement

The primary outcome was the change in HbA1c in patients with T2DM after 3 months of using Vitadio compared with an intraindividual control group. The secondary outcomes were changes in fasting glucose, body weight, and waist circumference. To determine the influence of Vitadio on quality of life, depression, and self-management, validated PRO questionnaires were collected. The Short Form Health Survey (SF-12) was used to measure changes in health-related quality of life [25,26]. Effects on self-management were evaluated using the Summary of Diabetes Self-Care Activities measure (SDSCA) [27], and effects on depression were measured by the Patient Health Questionnaire 9 (PHQ9) [28]. Additionally, patient adherence to the program and the effect of Vitadio on patient lifestyle were analyzed using app-reported data on diet and physical activity.

### 2.7. Sample Size

An a priori power analysis was performed using G*Power [29,30]. The expected change in HbA1c of 0.5% based on previous studies represents an effect size of 0.5 with SD (standard deviation) of 1% [19]. Considering a two-tailed *t*-test with a power and alpha of 80% and 5% respectively, the number of participants needed to detect the difference is 34. Because the attrition rate was unknown, a dropout rate of 40% was assumed, as in other studies examining digital therapeutics [31], leading to a sample size of 57.

### 2.8. Statistical Analysis

Data from the trial were analyzed to determine the impact of Vitadio use on changes in HbA1c values in patients with T2DM. Baseline data, i.e., demographic and amnestic data, were summarized. Continuous variables were examined using mean and standard deviation. Nominal variables were described using frequency distribution and chi-square test. All continuous parameters were tested for normal distribution using the Kolmogorov–Smirnov test. Correlations between parameters were examined using Pearson or Spearman correlation coefficients, based on the type of the underlying parameters.

Repeated measures ANOVA with the Bonferroni correction was applied to determine the overall significance of the differences in HbA1c values. A clinically meaningful effect was defined as HbA1c change >0.5% [32]. The differences in outcomes between Vitadio and standard diabetes care were determined by multiple post hoc tests. Missing HbA1c values from the retrospective control group were imputed with the mean of the remaining HbA1c values of the respective patient. Missing data from withdrawn patients were not imputed, as the CRFs with the HbA1c values are only sent at the end of the study and thus no HbA1c values for the patients are available. For other observed parameters (e.g., metabolic parameters, questionnaires, in-app data), a complete case analysis was performed. 

Changes in SF-12 and SDSCA scores as well as changes reported through in-app questionnaires were evaluated by paired *t*-tests or Wilcoxon signed-rank tests, as appropriate. The change in the number of patients falling into particular depression severity groups, defined by the PHQ-9 form, was assessed using the Stuart–Maxwell test. In addition, patient reported data from the app (food photos and daily steps) and app-generated data were analyzed, including retention, dropout rates, frequency of interactions, and adherence to the program provided through the app (e.g., average lesson reading time, achievement of personal goals, and adherence to self-monitoring). The methodology for app-data evaluation is described in particular in the results sections.

A linear regression model was employed to assess the effect of the app features on patient outcome, and relevant tests were used to check the validity of the model. Due to prevailing heteroscedasticity in one of the explanatory variables, coefficients are reported with robust standard errors.

Statistical significance was defined as a *p*-value < 0.05 (or 95% CI equivalent). All statistical analyses were performed using R Software version 4.0.3.

## 3. Results

### 3.1. Participant Characteristics

**Figure 2:**
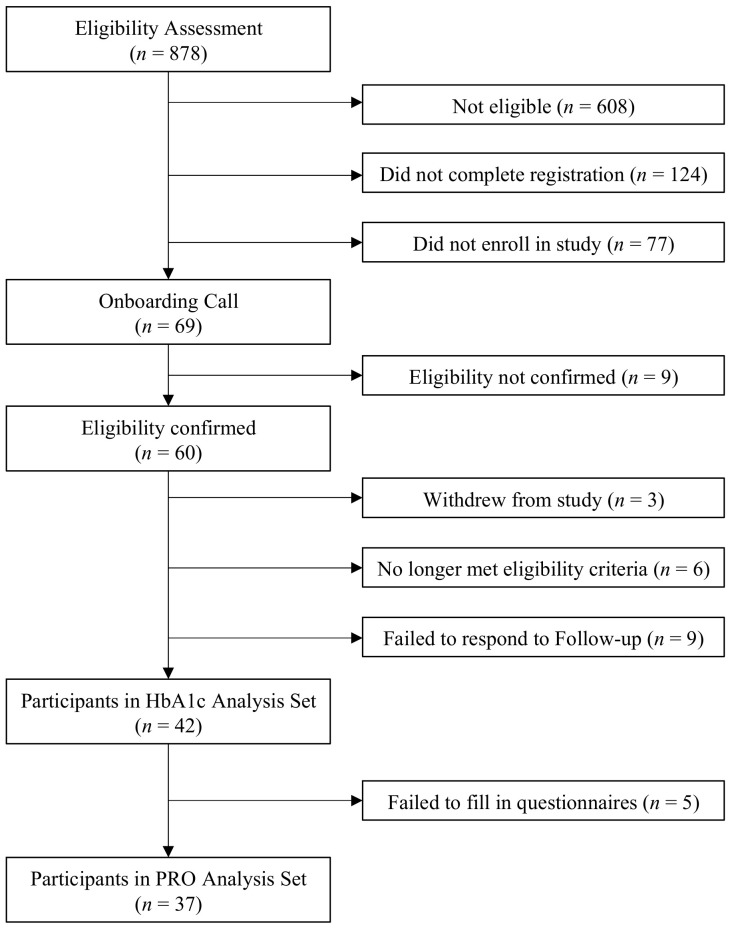
Study flow diagram. HbA1c = glycated hemoglobin, PRO = patient reported outcomes.

The entire process of eligibility assessment and enrolment in the study is illustrated in Figure 2. A total of 60 participants were enrolled in the study and of these, 42 (70%) participants submitted the HbA1c measurement at 3 months (end of study) and 37 (61%) completed the patient reported outcomes. The retrospective data from 3 months before the start of the Vitadio program were collected for 35 participants. The remaining seven data points were imputed by the mean value of all the remaining participant’s records.

On average, participants were 57 ± 7.4 years old and 55% male. Diabetes duration was reported by 26 participants, with an average time since diabetes diagnosis of 7.6 ± 6.4 years. Data on pharmacological treatment were obtained for all 60 participants: 37 participants were treated with peroral antidiabetics (PAD) only, 9 participants were treated with insulin therapy, and 14 participants were treated with a combination of insulin and PAD.

### 3.2. Effects on Glycemic Control

In the intervention group, the mean HbA1c baseline value was 7.9 ± 1.0%. After the 3 months of using Vitadio, the mean HbA1c value decreased on average by 0.9 ± 1.1% (*p* < 0.001), resulting in a 3-month follow-up value of 6.9 ± 0.9%. In the intraindividual control group, the mean HbA1c baseline value was 8.2 ± 1.3% (i.e., retrospective data from 3 months before start of using Vitadio) and the follow-up value was 7.9 ± 1.0% (i.e., the baseline value of the intervention group). The decrease of 0.3 ± 1.1% was not statistically significant (*p* = 0.27). The difference between the control and intervention groups was statistically significant [F (2, 78) = 28.26, *p* < 0.001], suggesting that intervention group receiving the Vitadio digital care achieved a clinically meaningful effect on lowering HbA1c and that these results appear up to be superior when compared to standard diabetes treatment. Table 1 summarizes the effect on glycemic control.

In addition, changes in HbA1c values were further examined based on various criteria. Participants under 55 years achieved a greater HbA1c reduction (−1.32 ± 1.25%) than participants older than 55 years (−0.78 ± 0.98%). Among male participants, HbA1c reduction was greater (−1.02 ± 1.1%) than among female participants (−0.83 ± 1.07%). However, both differences are not statistically significant. Participants with a baseline HbA1c value above 8 achieved a significantly (*p* < 0.001) greater HbA1c reduction (−1.61 ± 1.1%) than those with a baseline value below 8 (−0.43 ± 0.75%). Among participants with a baseline body mass index (BMI) over 30, a higher HbA1c reduction (−1.10 ± 1.11%) occurred than among participants with a baseline BMI under 30 (−0.6 ± 0.98%). The difference was not significant. * Metabolic parameters and HbA1c were recorded separately in the intervention group, resulting in a different sample size. Metabolic parameters were only recorded in the intervention group. Table 2 includes the subgroup analysis.

### 3.3. Effects on Metabolic Parameters

Metabolic parameters were only recorded in the intervention group. A significant reduction was identified in all four parameters: fasting glucose (baseline value: 7.4 ± 1.4 mmol/L, change: −0.6 ± 1.3 mmol/L), body weight (baseline value: 105.2 ± 18.5 kg, change: −4.3 ± 4.kg or 4% of the baseline bodyweight), BMI (baseline value: 35.1 ± 7.3 kg/m^2^, change: −1.4 ± 1.5 kg/m^2^), and waist circumference (baseline value: 121.1 ± 16.5 cm, change −5.7 ± 15 cm). The complete overview of metabolic parameters can be found in the Table 1.

### 3.4. Effects on Patient Reported Outcomes

The change in health-related quality of life and self-management was only reported by the intervention group. In total, both the baseline questionnaires and the 3-month questionnaires were collected from 37 subjects. The scores achieved in the individual questionnaires can be found in Table 3.

Changes in depression severity were not statistically significant. A shift between the moderate depression category (baseline: 5 subjects, 3-month: 1 subjects) was observed. Regarding quality of life, the Physical Component Summary (PCS) significantly increased and the Mental Component Summary (MCS) did not significantly increase. Minor trend improvements (i.e., in the category general diet and exercise) in adherence to self-management were observed by the SDSCA questionnaire, although the changes are not statistically significant.

### 3.5. Effects on App Reported Data

#### 3.5.1. Food Intake

Participants actively used meal photo logging, resulting in an average of 215 meal photos per participant and average daily self-evaluation of the dietary habits of 7/10. To assess the effect of Vitadio on dietary patterns, a graduate dietitian evaluated meal photos of selected participants from the first and last weeks. Participants with at least 10 inputs in the first week and 10 inputs in the last week were evaluated. Unrecognizable photos and text descriptions only were excluded from evaluation resulting in 531 meal photos from 24 participants (see Appendix B). Evaluation was based on an expert opinion and a methodology developed specifically for the purposes of the study, with each component being evidence-based and using a 1–5 Likert scale, with 1 being the best and 5 being the worst [33,34]. Following criteria were evaluated: portion size given the type of meal e.g., breakfast (1—adequate; 5—not adequate); amount of protein, carbohydrates, fiber, fruits and vegetables (1—adequate amount; 5—no source); the quality of fats (1—quality source; 5—too much fat or inferior source); the presence of ultra-processed foods or the adequacy of cooking technique (1—minimally-processed foods; 5—ultra-processed foods or inadequate technique); and the overall grade [35]. The evaluator was blinded to whether the photo belonged to the beginning or the end of the intervention. Significant improvements between the start and the end were observed across all evaluated categories. The detailed results are in Table 4.

#### 3.5.2. Physical Activity

For the analysis of the steps, data from 2 weeks at the beginning and 2 weeks at the end of the use of Vitadio were compared. To perform this analysis, the data were previously cleaned. To exclude an erroneous non-transmission, only days where the number of steps was over 500 were included. Users who recorded less than 500 steps on more than 30% of the days were completely excluded from the analysis. Only participants with valid records in both 2-week periods were included, resulting in 22 patients. The average number of daily steps over the whole 3 months was 6965 ± 3125, which is significantly higher (*p* = 0.02) than the German average for daily steps (5205) according to a Stanford study [36]. The number of daily steps increased from 6899 ± 3031 to 7200 ± 3346 after using Vitadio. However, the increase is not statistically significant (*p* = 0.23).

#### 3.5.3. Self-Management

Participants used various app features. They could choose 1 weekly habit from 23 predefined or self-created habits each week. Over 98% (*n* = 41) of participants set at least five habits over the 3-month period and 71% of participants set a new habit each week. On 65% of days, participants logged their habits as “achieved.” Over 96% of participants monitored their body weight and waist circumference at the recommended frequency or higher, and 80% of participants also used the app to monitor their blood glucose levels. Around 96% of participants completed all educational materials and 64% of participants completed all materials within the suggested weekly timeframe. 

A positive correlation (r = 0.42, *p* = 0.006) was found between compliance with habits and weight loss. Compliance with habits was defined as the share of days with logged habits (both successful and failed) in the total number of days with selected habits. In addition, success in diet-related habits correlated positively with weight loss (r = 0.47, *p* = 0.002) and with subjective disease management at 3 months (r = 0.46, *p* = 0.01). 

In total, 29 participants voluntarily measured their self-efficacy using in-app questionnaires covering nutrition, exercise, and diabetes management at baseline and 3-month follow-up of app use. A significant improvement in the ability to choose the right foods and be more physically active as well as subjectively perceived improvement in diabetes management were observed. The results are summarized in Table 4.

To determine the effect of each app feature on weight loss, a linear regression analysis was performed using app tracking data during the three months of Vitadio use. 

The effect of using selected app functionalities on weight reduction was examined. The evaluated features included weekly educational lessons, personal habits, and self-monitoring. Average time for reading one lesson, in minutes, was used as a proxy variable for attention paid to the education. Habit compliance, as defined above, was used to capture the adherence to daily routine. Total values for weight and waist were used as a proxy for adherence to self-monitoring. The model suggests that higher compliance with weekly habits, spending more time in the education section, and higher adherence to daily logging are associated with higher weight loss. Table 5 shows the results of the linear regression model. 

## 4. Discussion

The present study investigated the preliminary effectiveness of a digital lifestyle intervention on glycemic control, metabolic parameters, health-related quality of life, self-management, and depression. The major findings were that the therapy significantly reduced HbA1c and other metabolic parameters and improved quality of life after 3 months. At the baseline, only 21% of participants had an HbA1c value within the guideline-recommended target range of below 7.0% [13,37]. In the control group, the proportion of participants in the target range remained constant (17% of subjects at −3 months and 21% of subjects at baseline). After the 3-month intervention, the number of in-range patients increased such that 55% of participants were within the guideline-recommended target range. 

A recent review examined the evidence on the effectiveness of telehealth solutions and found that for digital self-management interventions using mHealth and involving lifestyle modification management, the average reduction in HbA1c was −0.52% [19]. In comparison, the Vitadio digital lifestyle intervention app was able to reduce HbA1c values by an average of −0.9% in this study. This review also found that subgroups with higher baseline HbA1c (>7.5% or >8.0%), patients younger than 55 years and with shorter time since diagnosis (<8.5 years and <7 years) had greater mean reductions in HbA1c. In this study, a significantly greater HbA1c reduction was shown for subgroups with a baseline HbA1c greater than 8%. In addition, a non-significant greater reduction was observed in patients younger than 55 years and with a baseline BMI of >30. The results of the study differ from the review only regarding the duration of diabetes, as a non-significantly greater HbA1c reduction was observed in participants with a duration of diabetes of more than 8.5 years. However, the duration of diabetes was a voluntary specification, so that only the data of 26 participants were evaluated, which is also reflected in the high standard deviation (SD = ±6.4 years). Further studies with larger sample sizes, longer follow-ups, and conducted under real-world conditions are needed to perform a more specific subgroup analysis and also with regard to increasing the efficacy of different diabetes DiGAs [38]. 

Several studies show that a 1% reduction in HbA1c results in a significant reduction in microvascular and macrovascular complications in patients with T2DM, targeting a HbA1c value below 7% to maximize the cardiovascular benefits [39,40,41].

If the results are sustainable, this app can potentially contribute to reducing cardiometabolic risk factors and to preventing T2DM. In the study, a significant reduction in BMI, weight and waist circumference were observed, which were associated with improvements in cardiometabolic risk factors [42]. Another risk factor for cardiovascular disease is physical inactivity [43]. In this study, participants reported an improvement in the physical activity scale measured by the SDSCA questionnaire, and there was a non-significant increase in the number of steps. However, more detailed research on the effects of the app on physical activity is needed. Inappropriate dietary composition and excess energy intake are other important factors affecting cardiovascular health [44]. We evaluated the change in dietary patterns of participants with emphasis on healthy behavior indicated by commonly accepted healthy-eating patterns, such as intake of whole grains, vegetables, fruits and lower intakes of saturated fats and ultra-processed food [45]. Participants achieved significant improvement across all evaluated criteria. The most prominent changes were observed in higher intakes of fiber, fruits, and vegetables. Through the linear regression model, it is estimated that the individual features of the app were associated with the following volumes of weight loss: One minute spent reading each of the educational lessons is related to 0.39 kg weight decrease, each percent of compliance with daily habits suggested 0.07 kg decrease, and each logging of either body weight or waist circumference suggested a decrease of 0.17 kg. The model explains 39% of the variability; other factors, apart from the specific app functionalities, enhancing the model performance could include socio-demographic factors, tracking relevant physical activity, or a change in dietary patterns. These could be investigated in further studies with adequate sample size.

In general, participants were very engaged with the app, with 85% using the app consistently throughout the 3 months (measured as interaction at least 2 days per week). An interaction was defined as at least 2 user actions within the app to exclude accidental opening of the app with no further user activity. Almost 30% of participants used the app daily (measured as at least 1 interaction every day) and 73% of participants used the app on an almost daily basis (measured as at least 1 daily interaction for at least 80% of days). This illustrates that Vitadio was well accepted by patients from Germany.

### Strengths & Limitations

Patients with a clinically relevant and realistic HbA1c spectrum between 6.5% and 11.0% were included to ensure that the study population is not over selected [46]. There was no interference with the patients, except for technical aspects, to obtain the most realistic observational evidence and to evaluate the effect of Vitadio on diabetes therapy under largely real-life conditions. To increase internal validity, the intervention period was scheduled between two physician visits to minimize uncontrolled confounding factors such as medication changes. In addition, different approaches were used including data from the app, questionnaires, and HbA1c levels. 

The present study had limitations such as the risk for selection bias, as motivated patients who are interested in apps and want apps as part of their treatment are more likely to participate in a study investigating a digital intervention. Recruitment was conducted via Facebook and participants received a financial reward of 30 EUR in the form of an Amazon voucher, which could also lead to selection bias. Other limitations are the small sample size and the self-reported data collection; further studies with larger sample sizes are needed to improve the validity and generalizability of the results. In addition, the data collection of the intraindividual control group was conducted retrospectively, resulting in seven participants with missing values, which were imputed due to the small sample size. The aim is not to generate any additional benefit in favor of the intervention group through missing values. As a full case analysis leads to a reduction in the number of participants and the number of participants is small (*n* = 42), a full case analysis was not considered useful here [47]. Due to the reduction in variability in the use of mean substitution, this method was used with caution and the results were critically questioned [48]. In addition, an intraindividual control group tends to overestimate the efficacy of the intervention [49]. Further studies with a parallelized control group and, to identify long-term effects, with longer follow-ups are planned [50]. Concomitantly, the study was not blinded, which is generally the case for digital interventions, so there is a risk of performance bias [51]. To reduce this particular source of bias, the use of a digital placebo can be considered and its robustness evaluated [52]. Furthermore, the results cannot be generalized to the German population as a whole, as not everyone owns a smartphone or a computer, and digital health literacy varies [38]. Further studies need to be conducted to evaluate the impact of digital health literacy [53]. 

The onboarding call of a dietician was offered not only to the study participants but also to the DiGA users to conduct the study under real-world conditions and minimize intervention bias. The meal photo analysis was performed by a dietitian from Vitadio, which may result in a bias. To minimize this risk, the dietitian was blinded to whether the photo was from the beginning or the end of the therapy. More detailed research is needed to investigate the influence of app features or subgroups on meal logging.

## 5. Conclusions

The findings of the present study demonstrate that a digital lifestyle intervention may be effective in lowering HbA1c, improving metabolic parameters, enhancing quality of life, improving dietary patterns, and increasing physical activity after 3 months. In addition, the findings support subgroup analysis possibly leading to a more targeted use of digital health applications and patient-centered treatment, although further research is needed in this regard.

## Figures and Tables

**Table 1:** Effects on glycemic control and metabolic parameters.

	Retrospective	Baseline	Follow-Up	Change	*p*-Value
	−3 months		+3 months		
*n* = 42 *					
HbA1c (%) Control group	8.2 ± 1.3	7.9 ± 1.0	-	−0.3 ± 1.1	0.27
HbA1c (%) Intervention group	-	7.9 ± 1.0	6.9 ± 0.9	−0.9 ± 1.1	<0.001
*n* = 37 *					
Intervention group					
Weight (kg)	-	105.2 ± 18.5	100.9 ± 17.6	−4.3 ± 4.5	<0.001
BMI (kg/m^2^)	-	35.1 ± 7.3	33.6 ± 7.1	−1.4 ± 1.5	<0.001
Waist circumference (cm)	-	121.1 ± 16.5	115.4 ± 17.4	−5.7 ± 15.0	0.03
Fasting glucose (mmol/L)	-	7.4 ± 1.4	6.8 ± 1.5	−0.6 ± 1.3	0.01

* Metabolic parameters and HbA1c were recorded separately in the intervention group, resulting in a different sample size. Metabolic parameters were only recorded in the intervention group.

**Table 2:** Subgroup analysis.

	Baseline HbA1c (%)	Follow-Up HbA1c (%)	Change in HbA1c (%)	*p*-Value	*p*-Value between Groups
*n* = 42					
<55 years (*n* = 12)	8.42 ± 0.95	7.09 ± 1.12	−1.32 ± 1.25	0.004	0.20
>55 years (*n* = 30)	7.66 ± 0.97	6.88 ± 0.78	−0.78 ± 0.98	<0.001	
*n* = 42					
Baseline HbA1c < 8% (*n* = 24)	7.17 ± 0.44	6.74 ± 0.76	−0.43 ± 0.75	0.01	<0.001
Baseline HbA1c > 8% (*n* = 18)	8.81 ± 0.77	7.21 ± 0.97	−1.61 ± 1.1	<0.001	
*n* = 42					
Baseline BMI < 30 (*n* = 12)	7.68 ± 1.05	7.07 ± 0.77	−0.6 ± 0.98	0.06	0.16
Baseline BMI > 30 (*n* = 30)	7.99 ± 1	6.89 ± 0.94	−1.10 ± 1.11	<0.001	
*n* = 26 *					
Duration < 8.5 years (*n* = 15)	7.6 ± 1.14	6.76 ± 0.88	−0.84 ± 1.31	0.008	0.58
Duration > 8.5 years (*n* = 11)	8.21 ± 0.64	7.1 ± 1.04	−1.12 ± 1.19	0.03	
*n* = 42					
Male (*n* = 23)	7.88 ± 0.98	6.86 ± 0.88	−1.02 ± 1.1	<0.001	0.57
Female (*n* = 19)	7.86 ± 1.08	7.04 ± 0.91	−0.83 ± 1.07	0.003	

* Diabetes duration was provided voluntarily, which resulted in the data of only 26 participants (61.9%) being analyzed.

**Table 3:** Patient reported outcomes.

	Baseline	Follow-Up	*p*-Value
		+3 months	
*n* = 37			
PHQ-9			
Depression severity (n)			0.36
Minimal	15	16	
Mild	13	15	
Moderate	5	1	
Moderately severe	3	3	
Severe	1	2	
SF-12			
PCS score	42.1 ± 9.6	45.4 ± 9.1	0.01
MCS score	42.1 ± 12.6	45.1 ± 13.6	0.06
SDSCA			
General Diet	5.3 ± 1.2	5.5 ± 1.3	0.30
Specific Diet	4.6 ± 1.5	4.5 ± 1.7	0.77
Exercise	3.7 ± 2.1	4.2 ± 1.8	0.10
Blood-Glucose Testing	4.7 ± 2.9	4.6 ± 2.9	0.50
Footcare	2.4 ± 2.4	2.3 ± 2.4	0.70
Overall Scale	4.1 ± 1.2	4.2 ± 1.2	0.47

**Table 4:** Effects on App Reported Data.

	Baseline	Follow-Up	Change	*p*-Value
		+3 months		
Meal Evaluation *				
*n* = 24				
Portion size	2.44 ± 0.40	2.18 ± 0.36	−0.26 ± 0.46	0.01
Protein	2.61 ± 0.56	2.28 ± 0.47	−0.32 ± 0.67	0.03
Carbohydrate	3.07 ± 0.51	2.68 ± 0.56	−0.38 ± 0.71	0.01
Fat	2.96 ± 0.69	2.59 ± 0.50	−0.37 ± 0.72	0.02
Fiber	3.33 ± 0.56	2.82 ± 0.54	−0.51 ± 0.63	<0.001
Vegetable	3.31 ± 0.92	2.67 ± 1.00	−0.64 ± 0.95	0.003
Processed food	2.10 ± 0.50	1.80 ± 0.43	−0.30 ± 0.50	0.007
Overall grade	3.08 ± 0.39	2.71 ± 0.36	−0.36 ± 0.42	<0.001
Self-efficacy				
*n* = 29				
In-app questionnaire **				
Ability to select proper food	4.86 ± 1.67	6.85 ± 1.74	1.99 ± 1.75	<0.001
Ability to be more active	6.45 ± 2.16	7.76 ± 2.42	1.31 ± 2.32	0.005
Diabetes management	5.89 ± 2.47	7.76 ± 2.42	1.89 ± 2.50	<0.001

* The evaluation was made based on a scale of 1–5. ** Participants answered the questions on a scale of 1–10.

**Table 5:** Linear regression model.

	Weight Change
Constant	8.286 (3.057) *
Lesson reading time	−0.392 (0.183) *
Habit compliance	−0.068 (3.341) *
Self-monitoring	−0.166 (0.028) **
Observations	41
R2	0.43
Adjusted R2	0.39

* *p*-value < 0.05; ** *p*-value < 0.001. Standard errors in parentheses. Values of weight change imputed from the app records for four participants.

**Figure A1:**
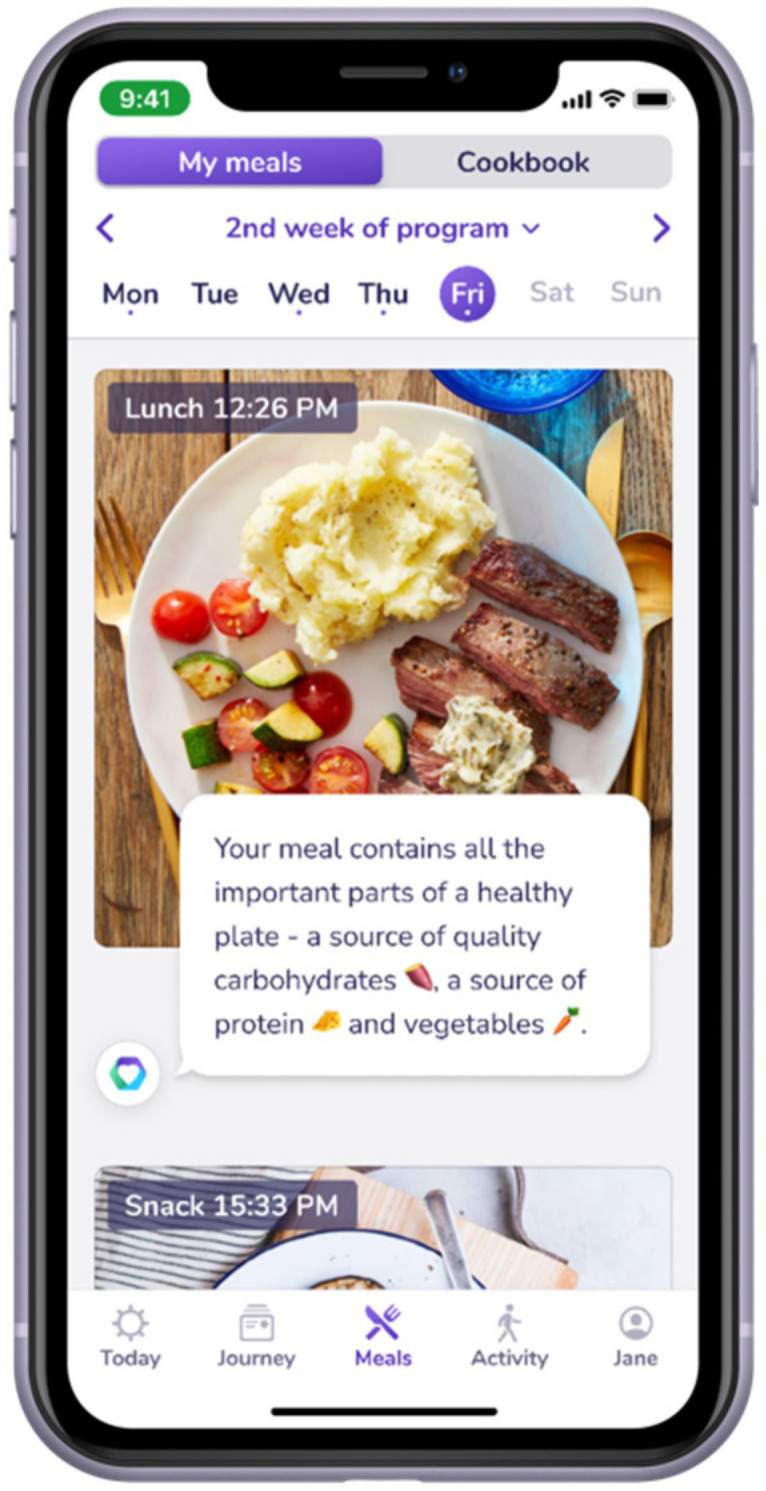
In-app meal logging function with automatic feedback.

**Figure A2:**
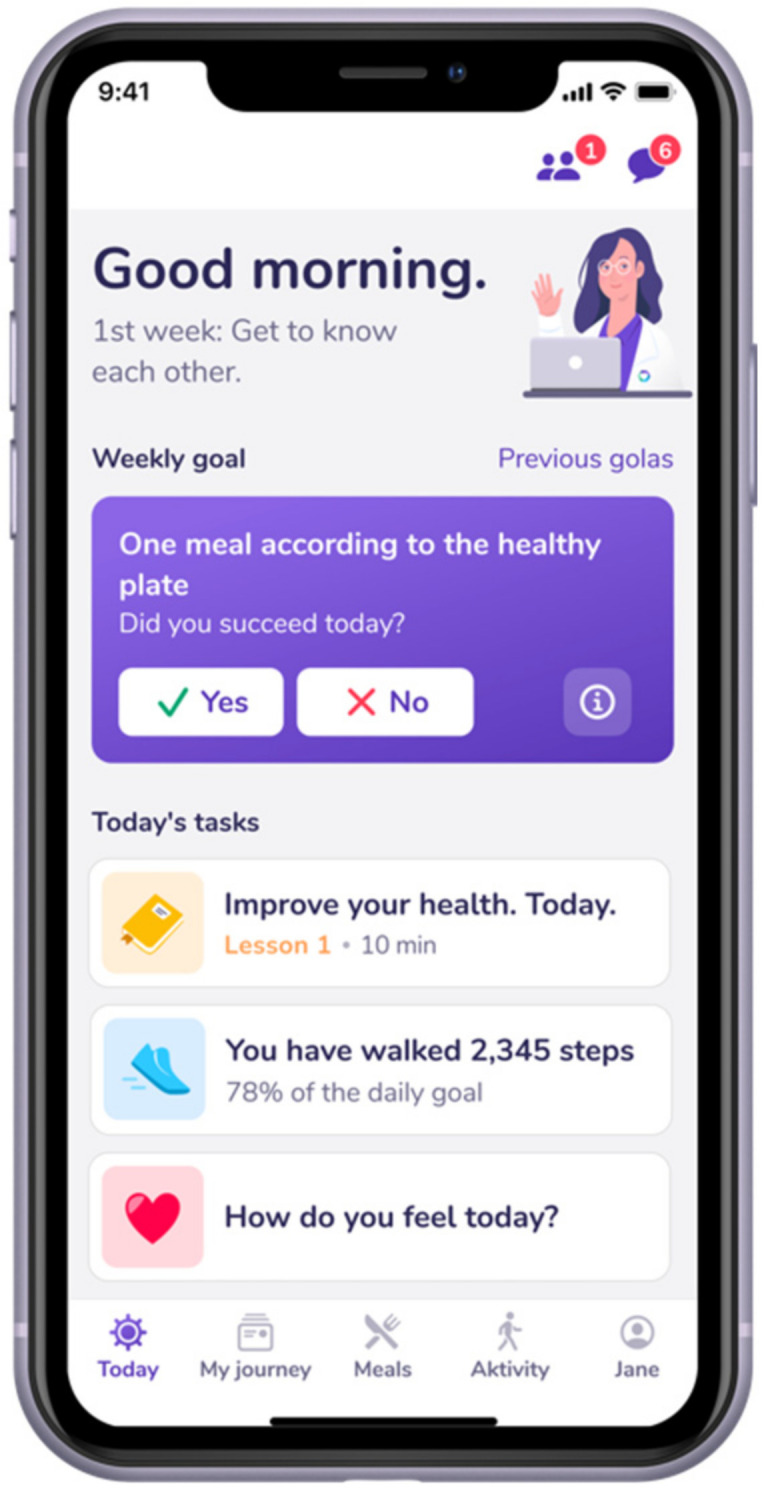
In-app home screen with daily tasks and personal goals.

**Figure A3:**
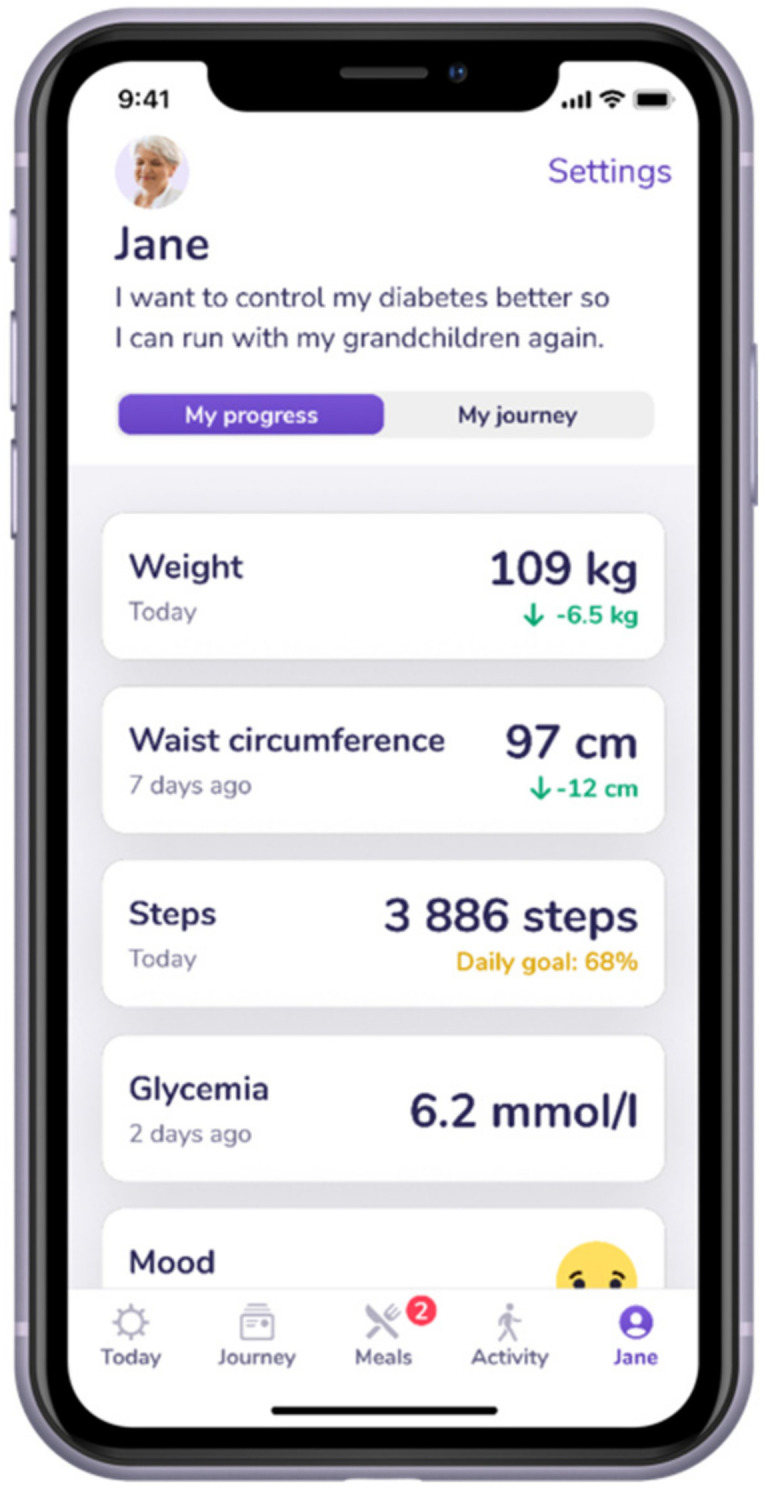
Tracking of health parameters.

**Figure A4:**
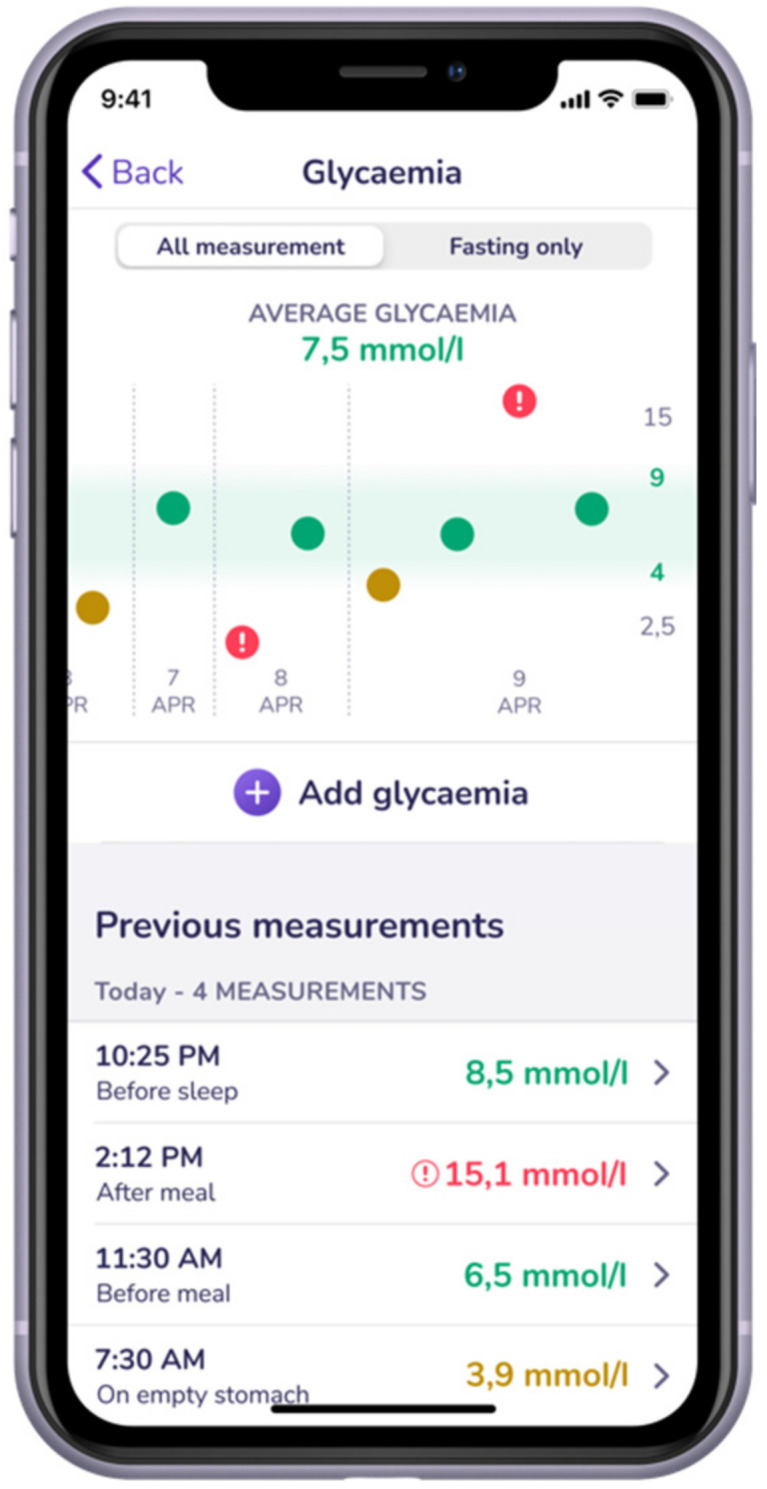
Glycemic values overview.

**Figure A5:**
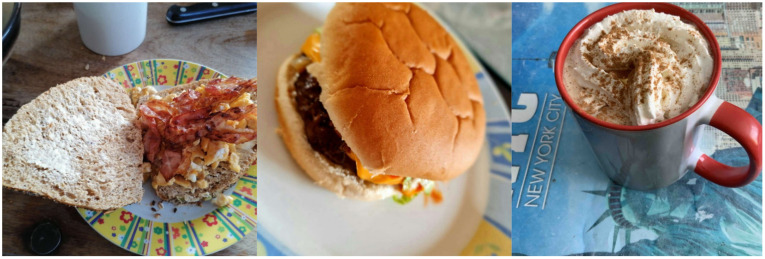
Meal photos at the start of the study.

**Figure A6:**
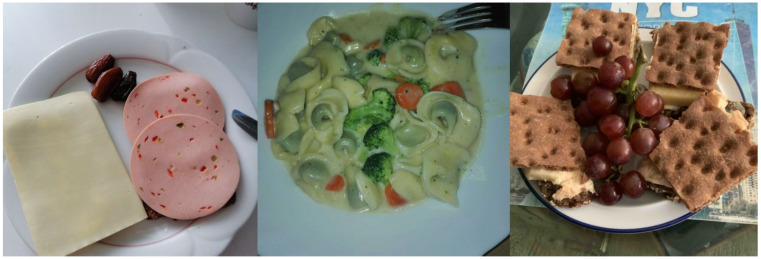
Meal photos at the end of the study.

## Data Availability

The data presented in this study are available to researchers who submit a methodologically sound proposal to the principal investigator, P.E.H.S. (peter.schwarz@uniklinikum-dresden.de). To gain access to the data, proposers will be required to sign a data access agreement.
